# An electrical characterisation methodology for identifying the switching mechanism in TiO_2_ memristive stacks

**DOI:** 10.1038/s41598-019-44607-3

**Published:** 2019-06-03

**Authors:** L. Michalas, S. Stathopoulos, A. Khiat, T. Prodromakis

**Affiliations:** Electronic Materials and Devices Research Group, Zepler Institute for Photonics and Nanoelectronics, University of Southampton, Southampton, SO17 1BJ UK

**Keywords:** Electrical and electronic engineering, Electronic and spintronic devices

## Abstract

Resistive random access memories (RRAMs) can be programmed to discrete resistive levels on demand via voltage pulses with appropriate amplitude and widths. This tuneability enables the design of various emerging concepts, to name a few: neuromorphic applications and reconfigurable circuits. Despite the wide interest in RRAM technologies there is still room for improvement and the key lies with understanding better the underpinning mechanism responsible for resistive switching. This work presents a methodology that aids such efforts, by revealing the nature of the resistive switching through assessing the transport properties in the non-switching operation regimes, before and after switching occurs. Variation in the transport properties obtained by analysing the current-voltage characteristics at distinct temperatures provides experimental evidence for understanding the nature of the responsible mechanism. This study is performed on prototyped device stacks that possess common Au bottom electrodes, identical TiO_2_ active layers while employing three different top electrodes, Au, Ni and Pt. Our results support in all cases an interface controlled transport due to Schottky emission and suggest that the acquired gradual switching originates by the bias induced modification of the interfacial barrier. Throughout this study, the top electrode material was found to play a role in determining the electroforming requirements and thus indirectly the devices’ memristive characteristics whilst both the top and bottom metal/oxide interfaces are found to be modified as result of this process.

## Introduction

Resistive Random Access Memories (RRAM) are two terminal devices that can support a multitude of resistive memory levels in a non-volatile fashion, triggered by an appropriate electrical stimulus^1,2^. This unique feature, also referred to as resistive switching (RS), along with the technogy’s potential to co-integrate RRAM cells with conventional semiconductor devices sparked a great interest in this field over the past decade. The potential of RRAM technology is thought to be fulfilled by enabling reconfigurable^3,4^ and neuromorphic systems^5,6^ towards establishing a new era in electronics technologies. The state-of-art in this field is also summarised in several topical reviews^7,8^. It is therefore timely to study and develop in more depth techniques and methodologies that allow us shining more light in the physical mechanism underpinning RS effects. This is essential for maturing this technology^9^ and to achieve performance optimisation and reliability towards realisation of commercial applications.

RS effects appear to depend upon various parameters including the active area material, the metal electrodes employed and the electroforming process that is typically required in most of the RRAM technologies reported to date. RS can be categorised in different ways. A macroscopic approach based on considering the polarity of the external stimulus that “sets” and “resets” a switching effect, referred to as bipolar or unipolar RS when opposite or identical polarity stimulus is respectively required^10^. If a more physics view is employed, RRAM technologies can be identified as (i) electrochemical metallisation cells (ECM) where RS relies upon the dissolution of an active electrode typically Ag or Cu^11^, (ii) valence change memories (VCM) where redox reactions lead to changes in the conductivity of the metal-oxide (MO) film^11^, (iii) thermochemical (TCM) in which RS is a result of a fuse/anti-fuse process due to current-induced temperature variation^11^ and (iv) interfacial, arising by the modification of the potential barrier at metal electrode/core film interface^12^.

While the nature of the switching mechanism can be directly studied by laborious physicochemical characterisation techniques^13^, indirect approaches employing electrical characterisation of RRAM prototypes can be rather beneficial due to their ease of use. This can be particularly useful in the case of interfacial mechanisms, where prompting the nature of nanoscale materials does not necessarily provide relevant information; certainly not with ease. RRAM devices typically exhibit a non-switching regime up to a certain threshold that onsets the switching process. When such technologies are operated within their switching regime, the nature of the RS mechanism has been reported to obey several operational characteristics such as abrupt or smooth/gradual transition from the High Resistive State (HRS) to the Low Resistive State (LRS), area dependent/independent current magnitude and linear/non-linear current voltage (I–V) characteristics to name a few. Recently, we demonstrated how the analysis of the I–Vs obtained at different temperatures can help with extracting signature plots that shine more light on the conduction mechanisms responsible for the transport at distinct resistive levels^14^. Inspired by this, the present paper introduces a methodology for extracting the switching mechanism nature of TiO_2_ RRAM cells by analysing the temperature dependence of their I–V characteristics in their non-switching regime, just before (after) a switching event takes place. Our methodology is validated with RRAM cells having common gold (Au) bottom electrodes (BE), identical TiO_2_ active area films and three distinct top metal electrodes namely gold (Au), nickel (Ni) and platinum (Pt). The top electrode (TE) material has been found to play important role on the electrical properties of these stacks in their pristine state^15^. Although hysteresis loops may be obtained in this state as well, they are more associated with mobile ions following the sweeping rate rather than with RS effects as those studied in this work. Nevertheless, the electrode materials may also affect even the post-electroforming^16^ electrical response of the device thus is a parameter to be considered.

## Methodology

**Figure 1:**
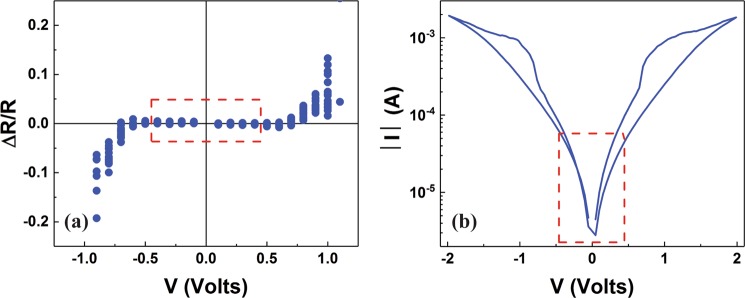
(**a**) Normalised change in the device resistive state as result of a sequence of applied pulses. The red rectangle defines the non-switching regime, i.e. the limit under which the stimulus bias does not affect the resistive state. (**b**) I–V curve showing RS behaviour. The non-switching regime (red rectangle) is defined by (**a**) and indicates where the analysis should be focused.

Application of electrical stimulus on RRAM cells onsets the RS effect. In order for this to take place and depending on the materials of the stack under investigation a threshold limit is needed to be reached. Identifying this limit that separates the switching from the non-switching operation regimes is the first step for our experimental methodology. This is performed by leveraging the capabilities of our in-house memristor characterisation platform ArC ONE^17^ and by following the algorithm described in details in^18^. Specifically, a sequence of programming pulses is applied and the normalised change in the resistive state is recorded after the application of a fixed number of pulses. Data resulting from this process are presented in Fig. 1(a), where apart from a bipolar RS type, it can be observed that a range of biases do not cause any change in the resistive state (highlighted by the red rectangle), defining the non-switching operation regime. An I–V curve is then recorded in a loop mode, eliciting stimulating pulses within the switching regime (Fig. 1(b)). Notwithstanding, this paper only focuses in analysing results acquired within the range of biases corresponding to the non-switching regime. We argue that analysing the mechanism responsible for the transport before the device reach the switching regime and just after this, remaining always in the non-switching area thus under equilibrium, it would be possible to conclude on the nature of the RS. As most of the conduction mechanism responsible for the transport in semiconductors and wide band gap materials exhibit both field and temperature dependence^19^, we opt recording the corresponding I–V curves of our samples over a range of temperatures. This allows separating between conduction mechanisms following similar dependencies^19,20^ and thus to minimise misinterpretations.

## Experimental Results

All samples employed in this work require an initial electroforming step in order to reveal their memristive character. Typically the electroforming of RRAM cells is performed by driving the device to a soft-breakdown and protecting them by using a current compliance^21^. This procedure has been successfully applied in similar stacks in the past^22^, however presently our electroforming protocol is based upon a pulsing-based, compliance free, procedure^14^. This is achieved by applying pulses of increasing amplitude and duration until the device resistance reaches a predetermined resistance level (Fig. S1 in the Supplementary Information). This protocol has been proven to be gentler with respect to the typical current compliance based one, possibly due to the ability of our characterisation instrument ArC ONE to respond promptly, i.e. minimising the induced damage. For this specific study and in order to be able to have some comparative outcomes, all prototype samples were formed by identical procedures with their parameters summarised in Table 1. As presented, all the devices can be formed using pulses having amplitudes ranges from 8 V to 12 V, but different durations are required for each one of them. Devices having Au TE require 1 µsec pulses for forming, while in the case of Pt or Ni TE, 500–750 µsecs and 1 msec are required respectively. This is in straightforward correlation with the interface barriers obtained on pristine devices^15^. More specifically, the lower the barrier in the pristine state the longer pulse duration is required to form the stack, possibly to its ability to adapt due to the higher conductivity. It is worth mentioning that the applied protocol allows for discrete devices to attain the same resistive levels, showing very similar I–V characteristics, particularly in the their non-switching operation regime (Fig. S2 in the Supplementary Information).

**Table 1:** Conditions of the pulsing-based compliance free protocol applied to the devices through ArC ONE.

	Au	Pt	Ni
Starting voltage (V)	8	8	8
Voltage step (V)	0.1	0.1	0.1
Up to ending voltage (V)	12	12	12
Number of pulses per bias	200	200	200
Pulse width (μsec)	1	500–750	1000
Series resistance	no	no	no

**Figure 2:**
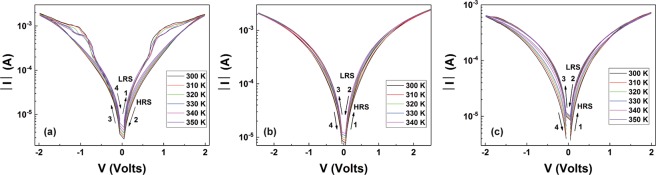
Extracting the transport properties requires recording of the I–Vs at different temperatures up to bias level that ensures RS. Three stacks with different TE studied in this work (**a**) Au/TiO_2_/Au, (**b**) Au/TiO_2_/Ni and (**c**) Au/TiO_2_/Pt. The arrows indicate the sweeping sequence, while the HRS/LRS levels should be considered only in the non-switching regime.

Following from this initial step, all the devices reached stable operation condition, showing clearly distinguishable resistive states (Fig. S3 in the Supplementary Information). These, apart from using the switching I–Vs sweeps are also attained successfully by stimulus pulses of the opposite polarity (Fig. S4 in the Supplementary Information). I–V characteristics were then recorded in the temperature range from 300–350 K that are presented in Fig. 2. Devices with Ni TE are breaking down at 350 K, thus the maximum operational temperature was 340 K. Using the acquired data, the transport properties for each I–V branches before and after each RS, for positive and negative bias polarities, were analysed, ensuring that the samples remain within their non-switching regimes by employing biases with amplitudes lower than 0.5 V. At this point it is important to clarify that the transport properties are strongly related to the operation temperature. Therefore our analysis provides related information for the applied range of temperatures only. Studies employing measurements in wider temperature range^23^ may reveal different character regarding the device electrical behaviour and/or even their ability for RS.

The Metal-TiO_2_-Metal stacks studied in this work can be equivalently modelled with a series combination of elements for the two interfaces and the active area, allowing us to extract the transport properties in their non-switching regimes. The overall dominant conduction mechanism is primarily determined by the most resistive one^15,24^. For a core-area dependent controlled transport (thus in the absence of interfacial barriers or if these are negligible), I–V curves should be symmetric with respect to the bias polarity. When the interfaces dominate the transport, asymmetric characteristics are expected^25^. This is a first indication useful for assessing the signatures supporting the dominant conduction mechanism. A second critical factor is the temperature dependence.

All the stacks studied in this work exhibit asymmetric and temperature activated characteristics. Considering the possible conduction mechanism^19^, this appears to be transport dominated by Schottky emission over the interfacial barriers.

For evaluating this it is essential to extract the so-called signature plots, which is a characteristic field and temperature dependence that uniquely defines a conduction mechanism. Schottky emission is described by Eq.^19^:

1 $$I=A{T}^{2}{e}^{-\frac{q({{\rm{\Phi }}}_{B0}-a\surd V)}{KT}}$$

Where A = (area × Richardson constant), *α* the barrier lowering factor, T the absolute temperature, K the Boltzmann constant, q the electron charge and Φ_B0_ the potential barrier at the interface under zero applied bias.

**Figure 3:**
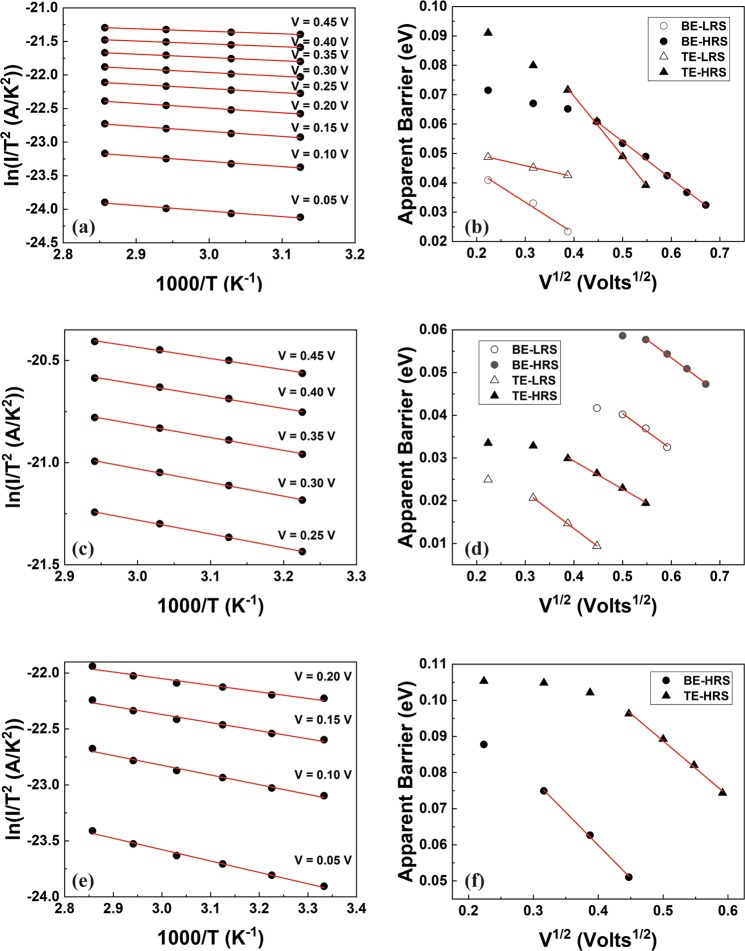
(**a**,**c**,**e**) Signature plots confirming the validity of Eq. 1 for the HRS branch correspond to the positive bias polarity of the I–V (see Supplementary Information for the signature plots correspond to the other I–Vs branches), thus supporting interface controlled transport, for Au, Ni and Pt TE respectively. (**b**,**d**,**f**) Additional signature plots generated by the previous ones and indicating modulation of the interfacial barrier further supporting the interface controlled transport for all the I–V branches of Au, Ni and Pt TE respectively.

Therefore, by plotting ln(I/T^2^) vs 1000/T, while ensuring applied biases maintain a non-switching regime, a linear relation is expected, where the slope corresponds to the apparent effective barrier (Φ_B0_ − *α*√V) under each specific electric field^15,24^, as depicted in Fig. 3(a,c,e). Moreover, we note that the apparent effective barrier should decrease by increasing the applied electric field as:

2 $${\rm{\Phi }}={{\rm{\Phi }}}_{B0}-a\surd V$$

and thus a plot of the apparent barrier calculated from the slope versus V^1/2^ should obeys a linear relation. If both these signatures are satisfied then the intercept of the last plot corresponds to the potential barrier at the interface under zero applied bias^15,24^ (Fig. 3(b,d,f)). Further to this and for the range of biases where the two plots described above are confirmed, bearing in mind Eq. 1, a straight line is also expected if the I–V curve at a specific temperature is plotted in ln(I) vs V^1/2^ plot (Figs S6–S9 in the Supplementary Information). The analysis for the different stacks is presented in Fig. 3 and at the Supplementary Information, supporting our findings for an interface-controlled transport in all cases. Regarding the LRS states in case of Pt TE, these appear to be temperature activated showing marginal asymmetry, also indicating an interface controlled transport but without being possible to extract clear signature plots. Such behaviour may be attributed to very low interface barriers and a boundary case towards a core material controlled transport (i.e. purely symmetric I–V).

## Discussion

**Figure 4:**
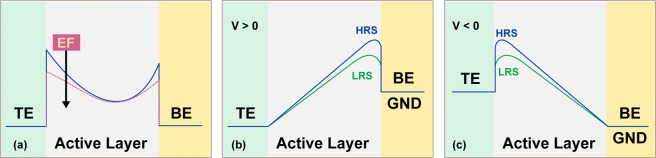
(**a**) A schematic diagram showing the potential barrier at the top and bottom interface for a pristine and an electroformed (EF) device. (**b**) Application of positive bias on the TE results in a forward biased Schottky contact at the top interface and a reverse biased one at the bottom interface. The barrier height is modulated during the sweep resulting in two different resistive states (LRS/HRS). (**c**) The situation is reversed when a negative bias is applied at the TE.

The analysis of the transport properties in the non-switching regimes, before and after switching effects, supports for all the cases studied in this work (all branches of the I–V curves) the existence of an interface controlled transport due to the presence of potential barriers at the TE or BE/TiO_2_ interfaces. Under this perspective the read-out resistance is a result of a reverse biased Schottky contact, corresponding to the bottom interface for positive biases and to the top one for negative polarities (Fig. 4).

Our analysis also revealed different barrier heights for the various branches of the I–V assessed in the non-switching regime, suggesting that driving the device through the switching regime results in modulation of the height for the dominant (each time) interfacial barrier; thus, the read-out resistance changes denoting an interfacial RS mechanism. This is also supported by the gradual “set” and “reset” processes in contrast to abrupt changes expected in cases where conductive filaments are formed and ruptured. The calculated barrier (Table 2) in this case is an effective one. Modifying this barrier by external electrical stimulus results in RS. Therefore for an interfacial type RS it is not necessary to obtain a uniform area dependence; although can be in support of this case. Moreover, the fact that the transport is controlled by the interfaces does not allow to further assess the properties of the core-film. Thus there is not enough evidence for commenting on possible changes in the MO microstructure as a result of the electrical stimulus (considering purely electrical measurements). Finally, considering Eq. (2) and the barrier lowering factor *α*, highlights the importance for the choice of the read-out voltage when characterising and implementing in circuits interfacial type devices.

**Table 2:** Post electroforming interface barrier heights extracted by the temperature analysis of the I–Vs in the non-switching regimes.

Φ_B0_ (eV)	BE	TE
***Au***		
HRS	0.117	0.151
LRS	0.065	0.057
***Ni***		
HRS	0.104	0.055
LRS	0.085	0.048
***Pt***		
HRS	0.133	0.164
LRS	Very low	Very low

Regarding the electrical stimulus and the reasons that modulate the barrier at the interface, this could stem from different origins. From the electronic point of view, modulation of the barrier is a result of changes in the charge existing at the interface and the depleted area of the reversed biased Schottky contact. This can be attributed to the migration of ions and/or cations^12,26^. Additionally, redox reaction^27^ may result in changes of the ionic concentration at the vicinity of the interface. A third reason could be electronic trapping/de-trapping at states existing at the interface or close to it. The latter one is a parameter that favours engineering towards bespoke modification of the barrier rendering interfacial RS devices as a potential candidates for applications require analogue behaviour. Separating these contributions to the interfacial barrier and particularly for an amorphous material it is not a straightforward task however, and requires thorough studies by multiple characterisation techniques applied in parallel.

Considering also the pristine state of the devices^15^ and the electroforming process, no straightforward correlation to the post-forming RS is revealed. Despite that, there are some interesting observations worth to be discussed. As a result of the electroforming process, both the top and bottom interfacial barriers are modified. Bearing in mind that in our case only positive polarity pulses applied, this indicates that it is most probably the film electronic properties rather than just the reverse biased interface that is affected by the forming protocol. These changes in the electronic properties of the film (e.g. by generated oxygen vacancies) result also in the reduction of the interfacial barriers at the post forming condition. Further to this and despite any straightforward correlation to the pristine barrier has not being revealed, the latter appears to determine the electroforming conditions, in our case the required pulse duration, and thus the post forming characteristics. Thus we may comment as a proof of concept evidence that proper selection of the TE metals may tune the forming process towards specific post-forming characteristics, although at the present state this should be considered as just a preliminary indication.

Finally we would like to add some thoughts regarding the I–V curves beyond the non-switching regime. Devices having Ni and Pt as TE, exhibit a typical bipolar character. As indicated in Fig. 1(a), devices with Au TE exhibit bipolar switching behaviour, as well, suggesting resistance increase when positively biased. However the shape of the I–V characteristic also resembles quite well that of complementary switching. Complementary RS takes place when two bipolar stacks are connected back to back^28^ and has been also demonstrated for single stacks having an internal metallic nanolayer^29,30^. This however is not the case for our stacks. We believe that understanding the electrical response of devices operate with interfacial RS mechanisms, outside the non-switching regime is not straightforward. The macroscopically obtained current conduction is this case is affected by the change in the dominant transport parameter from a reverse polarised Schottky to the opposite forward one (due to Schottky effect that diminishes the barrier) including effects of transient transports, due to trapping/de-trapping and/or ionic motions, notwithstanding the sweep characteristics. This is the major point of the proposed methodology. Understanding the induced modification required assessment in the non-switching operation regime just before and after switching occurs. Therefore the HRS/LRS should be considered only in this regime (Fig. 2).

## Conclusions

A methodology for revealing the nature of RS mechanism by analysing the transport properties of RRAM cells in their non-switching regime is presented. A pulsing based algorithm was initially applied in order to identify the switching/non-switching stimulus range. Afterwards the I–V characteristics were recorded and analysed at different temperatures considering both their field and temperature dependence along with additional features such as their symmetric/asymmetric response with respect to the applied bias polarity. The study presented in this work was focused on TiO_2_ based devices having identical Au BE, active area films and three discrete TE, Au, Ni and Pt. Signatures supporting transport controlled by the interfacial barriers extracted for all the cases, indicating RS due to bias induced modification of the interfacial barriers. Moreover although no straightforward correlation is obtained for the different TE or with respect to the pristine state interface barriers, there are some indications that this is critical for the electroforming requirements and thus might indirectly affect the device RS characteristics. Finally the features of interfacial type RS have also been discussed.

## Methods

### Device fabrication

The devices implemented in this work were fabricated on the same six-inch Si wafer having a 200 nm thick SiO_2_ layer that was grown by dry oxidation at 1000 °C with 5 slm O_2_ flow. All electrodes and active areas where patterned via standard optical lithography and liftoff processes^31^. A common BE metal was utilised, Au, with a thickness of 20 nm preceded by 5 nm Ti adhesive layer. The 24 nm active film, an amorphous TiO_2-x_, was deposited on top of Au BE by Lambda controlled plasma assisted reactive magnetron sputtering (Helios Pro XL, Leybold optics) using a Ti target, with 8 sccm O_2_, 35 sccm Ar flows and 2 kW at the cathode, and 15 sccm O_2_ flow and 2 kW at an additional plasma source. This industrial tool allows having high quality films with low thickness variations across the wafer. Using “Woolham Ellipsometer MC05” TiO_2-x_ thickness across six-inch wafer was found to be of 24.1 ± 0.27 nm. Films deposited by the above recipe exhibit an amorphous and almost stoichiometric nature of TiO_2-x_, with x in the range of 0.05–0.10, as previously demonstrated in the X-ray absorption spectroscopy study^22^. For the TEs, three separate areas were defined to deposit different TE metals: Au, Ni, and Pt, with thicknesses of 15 nm deposited by electron beam evaporation at a low rate of 0.5 Ås^−1^, comparable to deposition rate of the BE.

### Electrical characterisation

The current vs voltage (I–V) characteristics were obtained on 20 × 20 μm^2^ devices using our in-house memristor characterisation platform ArC ONE. The voltage sweeping was carried out always towards positive biases, while both positive and negative polarities were always applied to the TE with respect to the BE that was continuously kept grounded. All experiments were performed on a Cascade SUMMIT 12000B semi-automatic probe station that incorporates a thermal chuck, whose temperature can be controlled by an ESPEC ETC-200L unit. Measurements were performed in the temperature range of 300 K to 350 K. The discontinuity appearing at V = 0 V in some I–V curves is a result of our data acquisition system that does not acquire this point. This effect is negligible in most of the cases but not when measuring more conductive samples and for higher temperatures (340–350K) when the conductivity/current further increases. However this has no effect on the measured data. Finally it is worth mentioning the role of moisture which was reported to affect both the transport and switching properties of devices based on sputtered oxide films^32^. Bearing this in mind our experimental procedure was performed on environment where humidity and temperature were carefully controlled. Nevertheless this effect hasn’t shown significant influence in case of TiO_2_ layers^33^.

## Supplementary information

Supplementary Information

## Data Availability

All data supporting this study are openly available from the University of Southampton repository at: 10.5258/SOTON/D0930.

## References

[1] Kim W (2016). Multistate Memristive Tantalum Oxide Devices for Ternary Arithmetic. Sci. Rep..

[2] Stathopoulos S (2017). Multibit memory operation of metal-oxide Bi-layer memristors. Sci. Rep..

[3] Edwards AH (2015). Reconfigurable memristive device technologies. Proc. IEEE.

[4] Zidan M (2017). Field-Programmable Crossbar Array (FPCA) for Reconfigurable Computing. IEEE Trans. Multi-Scale Comput. Syst..

[5] Serb A (2016). Unsupervised learning in probabilistic neural networks with multi-state metal-oxide memristive synapses. Nat. Commun..

[6] Mehonic A, Kenyon AJ (2016). Emulating the electrical activity of the neuron using a silicon oxide RRAM cell. Front. Neurosci..

[7] Pan F, Gao S, Chen C, Song C, Zeng F (2014). Recent progress in resistive random access memories: Materials, switching mechanisms, and performance. Mater. Sci. Eng. R.

[8] Gao S (2019). Organic and hyvrid resistive switching material and devices. Chem Soc.Rev..

[9] Adam GC, Khiat A, Prodromakis T (2018). Challenges hindering memristive neuromorphic hardware from going mainstream. Nat. Commun..

[10] Waser R, Aono M (2007). Nonoionics-based resistive switching memories. Nat. Mater..

[11] Valov I (2017). Interfacial interactions and their impact on redox- based resistive switching memories (ReRAMs). Semicond. Sci. Technol.

[12] Yang JJ (2008). Memristive switching mechanism for metal/oxide/metal nanodevices. Nat. Nanotechnol..

[13] Yang, Y. & Huang, R. Probing memristive switching in nanoionic devices. *Nat. Electron*. **1**, 274–287 (2018).

[14] Michalas, L. *et al*. Conduction mechanisms at distinct resistive levels of Pt/TiO_2-x_/Pt memristors. *Appl. Phys. Lett.* **113**, 143503 (2018).

[15] Michalas L, Khiat A, Stathopoulos S, Prodromakis T (2017). Electrical characteristics of interfacial barriers at Metal - TiO_2_ contacts. J. Phys D: Appl Phys..

[16] Kim W-G, Rhee S-W (2010). Effect of the top electrode material on the resistive switching of TiO_2_ thin film. Microelectron. Eng..

[17] Berdan R (2015). A μ-Controller-Based System for Interfacing Selectorless RRAM Crossbar Arrays. IEEE Trans. Electron Devices.

[18] Serb A, Khiat A, Prodromakis T (2015). An RRAM Biasing Parameter Optimizer. IEEE Trans. Electron Devices.

[19] Sze, S. M. & Ng, K. K. *Physics of Semiconductor Devices*. (John Wiley & Sons, 2006).

[20] Michalas L, Koutsoureli M, Papandreou E, Papaioannou G (2013). Electrical Characterization of Undoped Diamond Films for RF MEMS Application. IEEE Intern. Reliabil. Phys. Sympos..

[21] Cao X (2009). Effects of the compliance current on the resistive switching behavior of TiO_2_ thin films. Appl. Phys. A Mater. Sci. Process..

[22] Carta D (2015). X-ray absorption spectroscopy study of TiO_2-x_ thin films for memory applications. J. Phys. Chem. C.

[23] Voronkovskii VA (2019). Conduction mechanisms of TaN/HfO_x_/Ni memristors. Mat. Res. Express.

[24] Michalas L (2018). Interface asymmetry induced by symmetric electrodes on metal-Al:TiO_x_-metal structures. IEEE Trans. Nanotechnol..

[25] Pintilie L, Vrejoiu I, Hesse D, LeRhun G, Alexe M (2007). Ferroelectric polarization-leakage current relation in high quality epitaxial Pb (Zr,Ti) O_3_ films. Phys. Rev. B - Condens. Matter Mater. Phys..

[26] Wedig A (2016). Nanoscale cation motion in TaO_x_, HfO_x_ and TiO_x_ memristive systems. Nat. Nanotechnol..

[27] Schönhals A (2018). Role of the Electrode Material on the RESET Limitation in Oxide ReRAM Devices. Adv. Electron. Mater..

[28] Linn E, Rosezin R, Kügeler C, Waser R (2010). Complementary resistive switches for passive nanocrossbar memories. Nat. Mater..

[29] Gao S (2015). Implementation of Complete Boolean Logic Functions in Single Complementary Resistive Switch. Sci. Rep..

[30] Gao S (2015). Tuning the switching behavior of binary oxide- based resistive memory devices by inserting an ultra-thin chemically active metal nanolayer: a case study on the Ta_2_O_5_ – Ta system. Phys. Chem. Chem. Phys..

[31] Serb A, Khiat A, Prodromakis T (2018). Seamlessly fused digital-analogue reconfigurable computing using memristors. Nat. Commun..

[32] Lübben M, Wiefels S, Waser R, Valov I (2017). Processes and Effects of Oxygen and Moisture in Resistively Switching TaO _*x*_ and HfO _*x*_. Adv. Electron. Mater..

[33] Tsuruoka T (2012). Effects of moisture on the switching characteristics of oxide-based, gapless-type atomic switches. Adv. Funct. Mater..

