# Supplementary material for: An electrical characterisation methodology for identifying the switching mechanism in TiO2 memristive stacks
Source: Sci Rep. 2019 Jun 3;9:8168. doi: 10.1038/s41598-019-44607-3 (PMC6546741; doi:10.1038/s41598-019-44607-3)
Supplement: Supplementary file 1 — Supplementary Information [file 41598_2019_44607_MOESM1_ESM.pdf]

Supplementary information

An electrical characterisation methodology for identifying the switching mechanism in TiO_2 memristive stacks

L. Michalas*, S. Stathopoulos, A. Khiat and T. Prodromakis

Electronic Materials and Devices Research Group, Zepler Institute for Photonics and Nanoelectronics,

University of Southampton, SO17 1BJ, UK

*Corresponding Author: l.michalas@soton.ac.uk

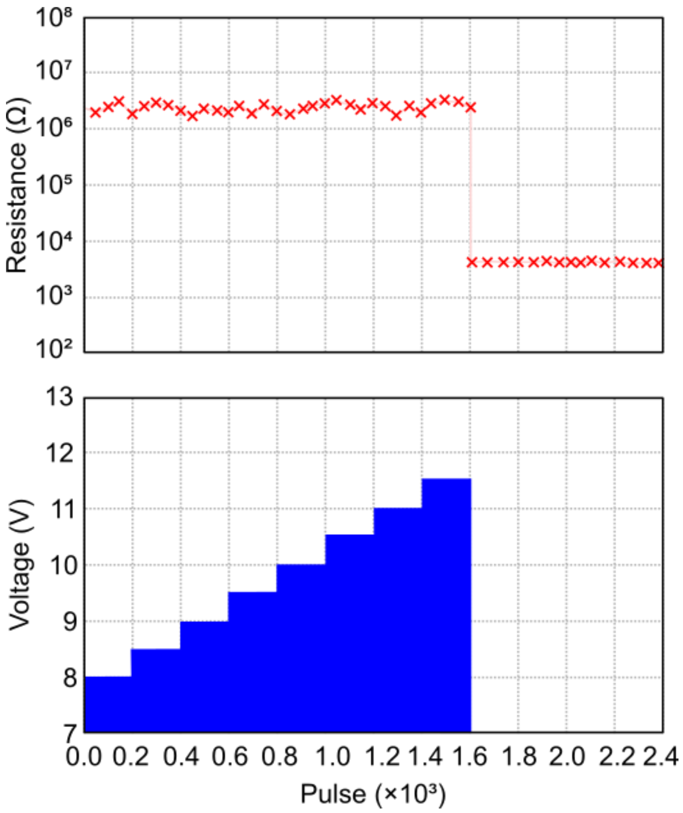

Figure S1: Representation of our pulsing based and compliance free electroforming protocol. This is based on applying trains of pulses having specific time-width and progressively increasing amplitude, as summarized in table I.

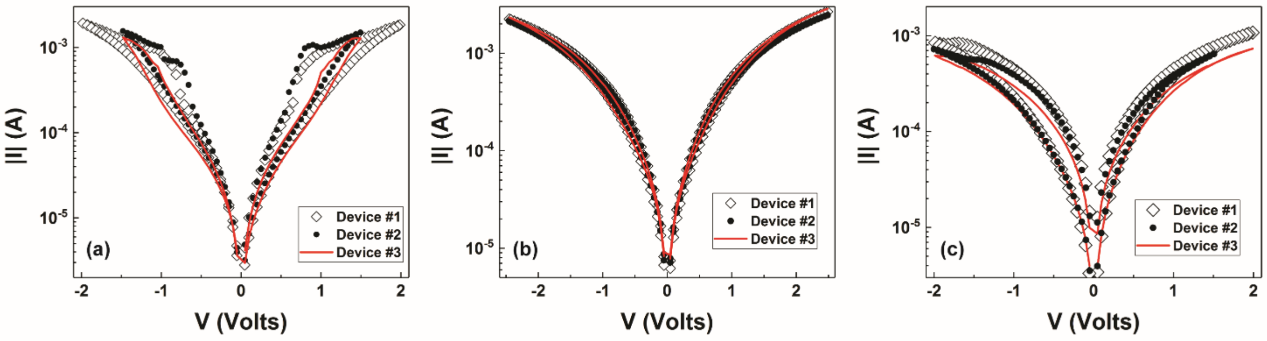

Figure S2: Our electroforming protocol allows for driving discrete devices of each stack, with (a) Au TE, (b) Ni TE and (c) Pt TE, on the same resistive levels, showing very similar I-V characteristics, in particularly in the their non-switching operation regime.

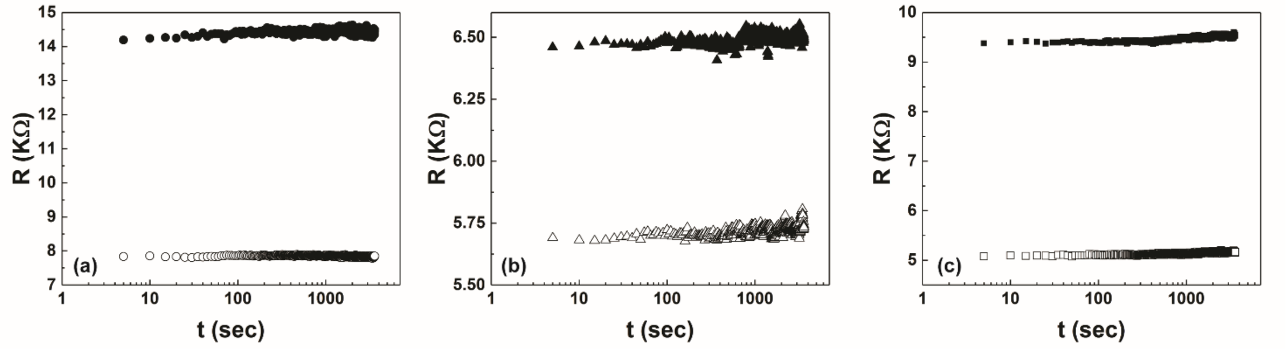

Figure S3: Retention characteristics of devices having (a) Au TE, (b) Ni TE and (c) Pt TE, showing stable and clearly distinguishable low and high resistive states.

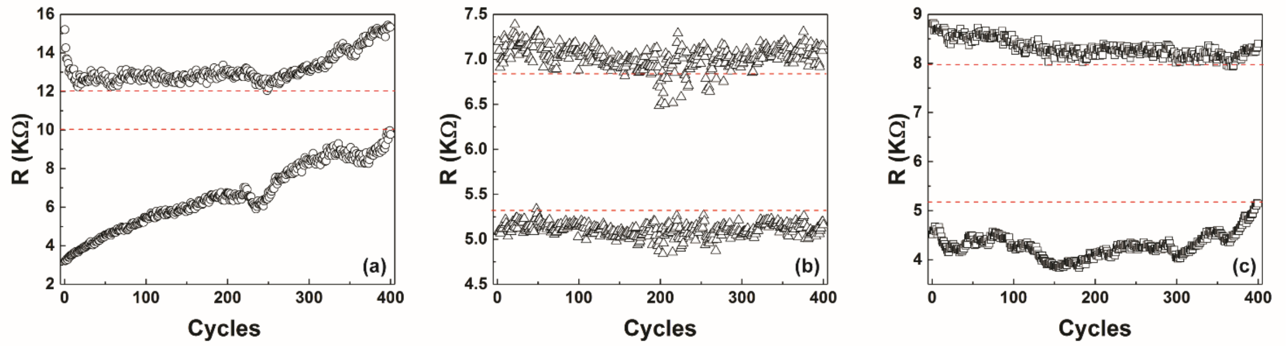

Figure S4: Indicative bipolar switching ability induced by single pulses of opposite polarities (endurance). All stacks respond successfully supporting their bipolar switching character. A drift is observed on devices with Au TE (a), maintaining however the bipolar character and a clear window up to 400 switching cycles. Devices having Ni (b) and Pt (c) TE exhibit more stable switching characteristics.

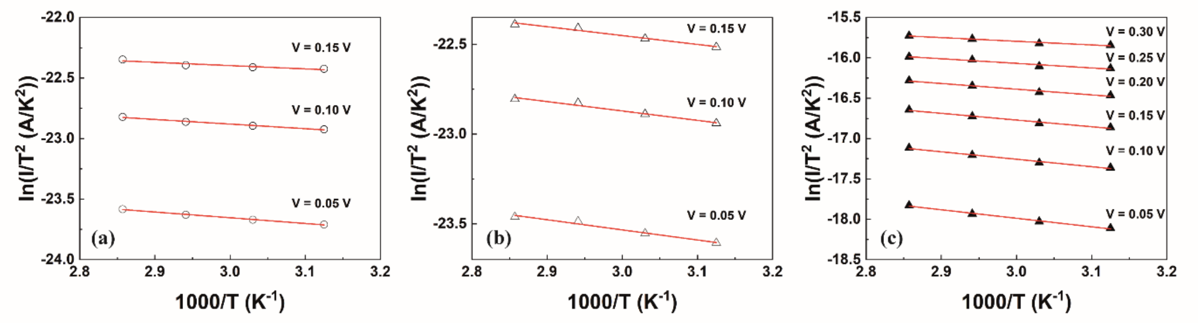

Figure S5: Signature plots confirming the validity of Eq. 1, for the LRS branch of the I-Vs correspond to positive biases (a) and for the LRS (b) and HRS (c) branches correspond to negative applied biases, for devices having Au TE (Figure 2(a)). The effective apparent barriers calculated from the slopes are those presented in Figure 3(b), with empty and filled symbols respectively.

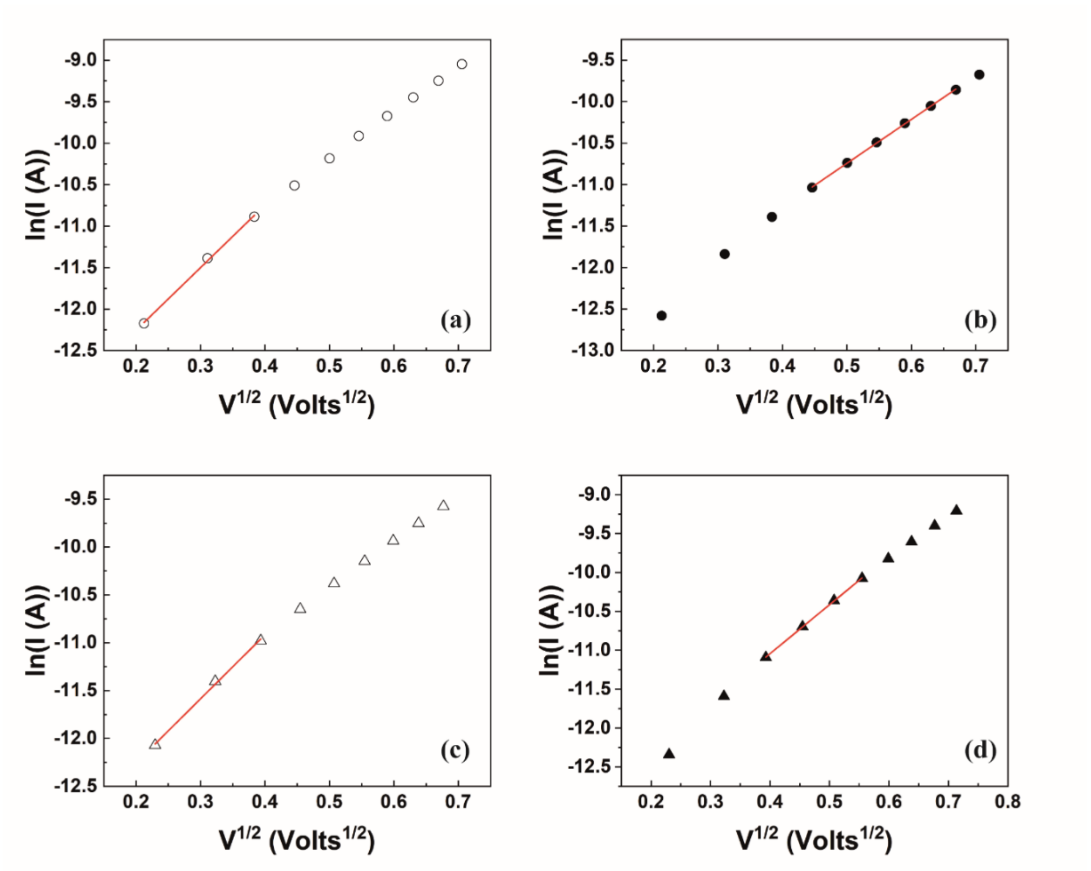

Figure S6: Constant temperature signature plots further confirming the validity of Eq. 1 for devices having Au TE and for all the branches ((a)-(d) same symbols) of the I-Vs (figure 2(a)) in the range of the applied biases indicated by the temperature dependent signature plots in Figure 3(b).

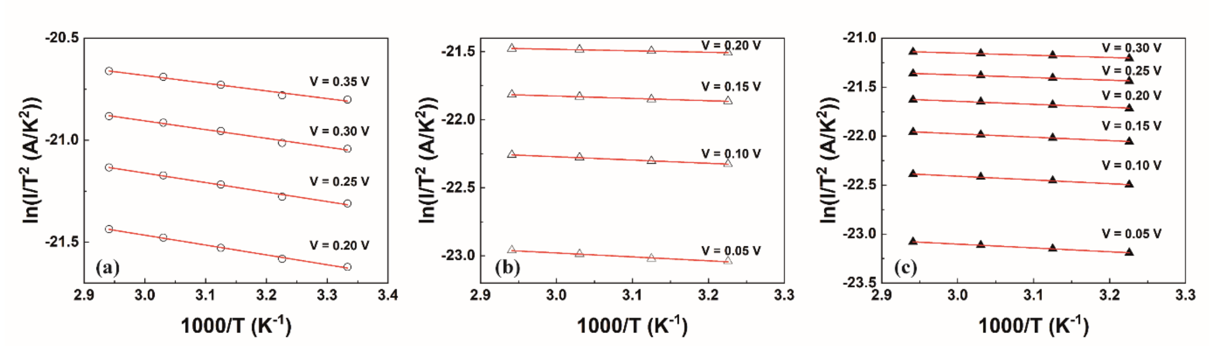

Figure S7: Signature plots confirming the validity of Eq. 1, for the LRS branch of the I-Vs correspond to positive biases (a) and for the LRS (b) and HRS (c) branches correspond to negative applied biases, for devices having Ni TE (Figure 2(b)). The effective apparent barriers calculated from the slopes are those presented in Figure 3(d), with empty and filled symbols respectively.

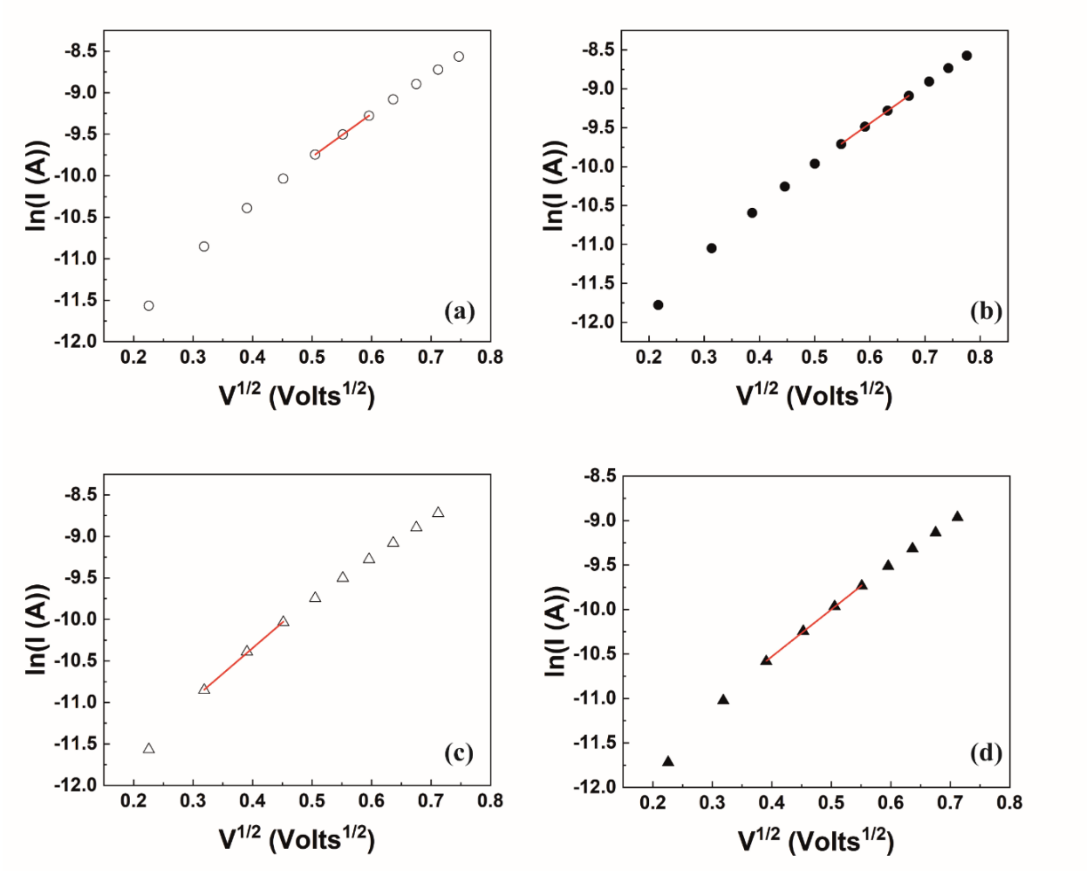

Figure S8: Constant temperature signature plots further confirming the validity of Eq. 1 for devices having Ni TE and for all the branches ((a)-(d) same symbols) of the I-Vs (figure 2(b)) in the range of the applied biases indicated by the temperature dependent signature plots in Figure 3(d).

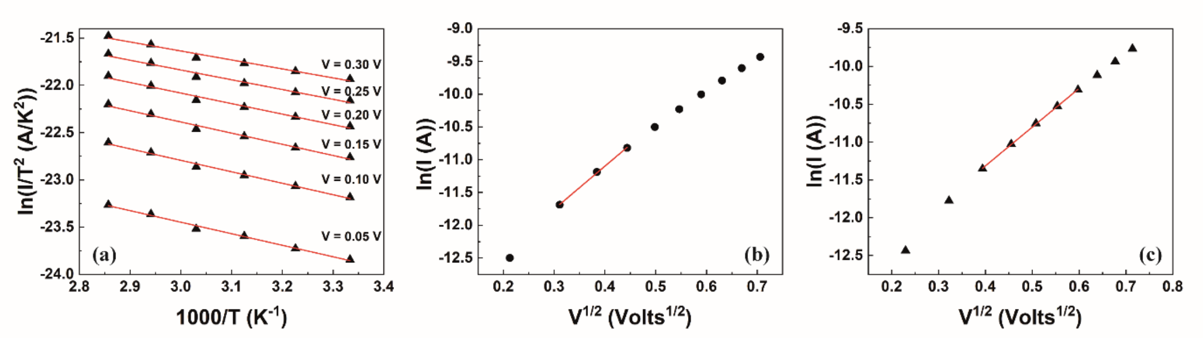

Figure S9: Signature plots confirming the validity of Eq. 1, for the HRS branch of the I-Vs to negative applied biases (a), for devices having Pt TE and constant temperature signature plots further confirming the validity of Eq. 1 for devices having Pt TE and for the two HRS branches ((b)-(c) same symbols) of the I-Vs (figure 2(c)) in the range of the applied biases indicated by the temperature dependent signature plots in Figure 3(e).
